# Lignocellulolytic Enzymes Production by Four Wild Filamentous Fungi for Olive Stones Valorization: Comparing Three Fermentation Regimens

**DOI:** 10.4014/jmb.2312.12048

**Published:** 2024-03-25

**Authors:** Soukaina Arif, Hasna Nait M’Barek, Boris Bekaert, Mohamed Ben Aziz, Mohammed Diouri, Geert Haesaert, Hassan Hajjaj

**Affiliations:** 1 Moulay Ismail University of Meknès, Laboratory of Biotechnology and Bioresources Valorization, BP 11201, Zitoune Meknes City, Morocco; 2 Moulay Ismail University of Meknès, Cluster of Competency «Agri-food, Safety and Security» IUC VLIR-UOS, Marjane 2, BP 298, Meknes City, Morocco; 3 Paris-Saclay University, CentraleSupélec, European Center of Biotechnology and Bioeconomy (CEBB) - LGPM, 3 rue des Rouges Terres, 51110 Pomacle, France; 4 Ghent University, Faculty of Bioscience Engineering, Department of Plants and Crops, Valentin Vaerwyckweg 1, Schoonmeersen - gebouw C 9000 Ghent, Belgium; 5 Sultan Moulay Sliman University, Higher School of Technology, Laboratory of Biotechnology, Bioresources, and Bioinformatics (3BIO), 54000 Khenifra, Morocco

**Keywords:** Lignocellulolytic enzymes, filamentous fungi, sequential solid-state and submerged fermentation, olive stones, biorefinery

## Abstract

Lignocellulolytic enzymes play a crucial role in efficiently converting lignocellulose into valuable platform molecules in various industries. However, they are limited by their production yields, costs, and stability. Consequently, their production by producers adapted to local environments and the choice of low-cost raw materials can address these limitations. Due to the large amounts of olive stones (OS) generated in Morocco which are still undervalued, *Penicillium crustosum*, *Fusarium nygamai*, *Trichoderma capillare*, and *Aspergillus calidoustus*, are cultivated under different fermentation techniques using this by-product as a local lignocellulosic substrate. Based on a multilevel factorial design, their potential to produce lignocellulolytic enzymes during 15 days of dark incubation was evaluated. The results revealed that *P. crustosum* expressed a maximum total cellulase activity of 10.9 IU/ml under sequential fermentation (SF) and 3.6 IU/ml of β-glucosidase activity under submerged fermentation (SmF). *F. nygamai* recorded the best laccase activity of 9 IU/ml under solid-state fermentation (SSF). Unlike *T. capillare*, SF was the inducive culture for the former activity with 7.6 IU/ml. *A. calidoustus* produced, respectively, 1,009 μg/ml of proteins and 11.5 IU/ml of endoglucanase activity as the best results achieved. Optimum cellulase production took place after the 5th day under SF, while ligninases occurred between the 9th and the 11th days under SSF. This study reports for the first time the lignocellulolytic activities of *F. nygamai* and *A. calidoustus*. Furthermore, it underlines the potential of the four fungi as biomass decomposers for environmentally-friendly applications, emphasizing the efficiency of OS as an inducing substrate for enzyme production.

## Introduction

Worldwide, olive oil production has increased threefold in the previous 60 years, reaching 3,098,500 tons in the 2021/22 crop year, as estimated by the International Olive Council [1]. This sector generates an enormous quantity of by-products with a noticeable environmental footprint and applications with less added value each year. Representing about ten percent of the weight of the olive fruit, the olive stones (OS) are the solid residue of the olive oil extraction process. They are obtained in the form of small, crushed fragments after the separation of the remaining pulp and a secondary extraction of the residual oil [2, 3]. The primary application of this biomass is in the generation of electric power or heat through combustion. Recently, it has drawn the attention of researchers because of its valuable composition rich in lignocellulose (LC), namely, cellulose, hemicellulose, and lignin [4], phenols, fats, proteins, and free sugars [5]. After processing, this by-product, also called olive pits or kernels, can serve as a bioenergy feedstock [6], animal feed [7], and nutritional supplement [8], in the cosmetic and pharmaceutical industries [9, 10]. It can also be employed as a precursor to produce activated carbon to be used as an adsorbent [11].

In the biorefinery concept, OS are recognized as a type of LC biomass with significant potential for the production of fuels and bio-chemicals, thus being a promising raw material [12, 13]. Green and safe processes such as enzymatic techniques are used to ensure the efficient exploitation of this valuable biomass. Enzymatic lignocellulose decomposition, a process that consists of converting LC into oligomeric and monomeric units, the substrate complexity requires the use of specialized enzymes [14]. These enzymes are mainly composed of cellulases, hemicellulases, ligninases, and auxiliary enzymes responsible for the degradation of the plant cell wall constituents [15]. So far, many lignocellulolytic enzymes produced by microorganisms, have been identified and clustered into various families in the Carbohydrate-Active EnZyme database (CAZyme), taking their sequence and structural-functional characteristics as a basis for their classification [16].

In the natural environment, fungi stand out among microorganisms due to their remarkable proficiency in degrading LC. This proficiency stems from their capacity to release a wide array of hydrolytic and oxidative extracellular enzymes, which are designed to selectively target the distinct constituents of plant cell walls [17]. Three fungal groups responsible for lignocellulose depolymerization have been described: white‐rot, brown‐rot, and soft‐rot fungi [18]. The cellulose degradation process is performed by glycoside hydrolase (GH) enzymes that possess synergistic catalytic activity. Cellulases are classified into three prevalent groups: endoglucanases (EG)(endo-1-4-β-glucanase; EC 3.2.1.4), exoglucanases (EC 3.2.1.91) and β-glucosidases (EC 3.2.1.21) in charge of β-1,4-glycosidic linkage hydrolysis between glucose units within the cellulose fiber [19]. Hemicellulose hydrolysis requires essentially endo‐1,4‐β‐xylanases (EC 3.2.1.8) for the generation of oligosaccharides, and exo‐1,4‐β‐xylosidase (EC 3.2.1.37) that release xylose by cleavage of oligosaccharides [18]. In terms of lignin consumption, the main groups of ligninolytic enzymes involved include laccases (EC 1.10.3.2; CAZy AA1), manganese-dependent peroxidases (EC 1.11.1.13; CAZy AA2), lignin peroxidases (EC 1.11.1.14; CAZy AA2) and versatile peroxidases (EC 1.11.1.16; CAZy AA2) [20]. Generally, the lignin degradation process is accomplished by white‐rot fungi as secondary metabolism, or due to the limited disposal of carbon, nitrogen, or sulfur [21]. Nowadays, lignocellulolytic enzymes are largely used in the food industry for various applications, such as product texturizing and flavoring, particularly during the processing of fruits and vegetables [22]. Additional areas of interest include biorefinery, textiles, animal feed, and many other sectors [23].

The fermentation process is among the main conditions that influence microbial growth, enzymatic expression, and productivity [24, 25]. Many fermentation regimens have been described representing both benefits and barriers. Solid-state (SSF) and submerged fermentations (SmF) are the common conventional processes widely employed for lignocellulolytic enzyme production. The first one offers remarkable strengths regarding the suitability of LC biomass as a substrate under growth requirements closely reflecting the natural environment in which fungi are adapted. This positive alignment leads to better microbe/ substrate interaction reflected by greater enzyme titers. Nevertheless, the SSF presents complexities in terms of control in scaling-up processes [26, 27]. In comparison, SmF shows great promise. The presence of established methods for monitoring and controlling bioreactors makes it the favored approach for industrial enzyme production. Despite yielding more diluted products, the outcome of the SmF process can be directly utilized as enzyme cocktails by various industries [28, 29]. Based on the limitations of these two methods, sequential fermentation (SF), also called sequential solid-state and submerged fermentation, has been developed as an economical and successful approach. This approach involves using LC as an induction substrate for pre-culture preparation, which is achieved by an SSF step followed by a switch to SmF. Combining the advantages of the two techniques, the process promotes greater enzyme production and improves both substrate assimilation and fungal growth morphology [30-32].

*Penicillium*, *Fusarium*, *Trichoderma*, and *Aspergillus* genera belonging to the Ascomycota division, are well-known filamentous fungi capable of producing extracellular enzymes. Their crucial role in efficiently breaking down complex plant cell wall components has been extensively studied and optimized at different scales [33-36]. However, the development and widespread industrial use of lignocellulolytic enzymes face several limitations and challenges. These include their high cost, stability, and specificity, and the wide variety of biomasses used. Therefore, there is a growing focus on identifying new, more cost-effective enzyme sources and applying advanced technologies to overcome these obstacles. This effort aims to capitalize on the potential of these enzymes across different biomasses and in various industrial applications [37-39].

In Morocco, according to official reports from the Ministry of Agriculture, the olive tree dominates the culture of fruit species, accounting for 65% of the national tree area [40]. The olive oil sector is a flagship field in the Fez Meknes region, one of the two regions in the kingdom where 54% of the olive oil area is concentrated. Olive oil processing generates a significant quantity of by-products, such as OS, which represent an impressive source of bioconstituents. Previous research [41] revealed that this by-product collected from our study region, exhibited a significant oxygen content of approximately 35.66 ± 0.71%, indicating its recalcitrant nature. Besides, OS have attracted researchers’ interest due to their valuable composition rich in LC materials, including cellulose (27.1-36.4%), hemicellulose (24.5-32.2%), and lignin (26.0-40.4%) [42], making them promising sources for enzyme production.

This study aims to explore the potential of local biomass and wild fungal species in green bioprocesses that require further attention and consideration at the national level. *Penicillium crustosum*, *Fusarium nygamai*, *Trichoderma capillare*, and *Aspergillus calidoustus* were selected for their diversity in lignocellulolytic activities in our previous work [43]. The other objective is to investigate the most appropriate fermentation system for the production of lignocellulolytic enzymes. The use of OS as a substrate is a significant feature to encourage the implementation of biorefineries in the region of Fez-Meknes due to the large availability of this by-product. On the one hand, the enzymatic cocktail that can be produced represents a highly promising market. On the other hand, the bioconversion of this by-product into high-value-added molecules can provide a significant economic impact on the national olive sector.

## Materials and Methods

### Culture Media and Chemicals

All chemicals used in this study were supplied by Sigma Aldrich or Thermo Fisher Scientific (ACS grade, France). The high-purity enzyme substrates (≥ 95%) employed in enzymatic assays include 1^st^ grade Whatman filter paper (WHA1001150), carboxymethyl cellulose (CMC; 419311-100G), cellobiose (C7252-25G), 2,2-azino-bis (3-ethylbenzothiazoline-6-sulfonic acid) (ABTS; A1888-5G), veratryl alcohol (VA; D133000-25G) and phenol red (114529-5G).

Commercial Potato Dextrose Agar (PDA) was used for in-plate fungal growth, subculturing, and conservation. For fermentation experiments, an adapted medium from Mendels & Sternberg [44] was used with the following composition: peptone; 0.5%, yeast extract; 0.2%, KH_2_PO_4_; 0.2%, (NH_4_)_2_SO_4_; 0.14%, Tween 80; 0.1%, salt solution; 0.1% (FeSO_4_·7H_2_O; 5 mg/l, CoCl_2_; 2 mg/l, MnSO_4_·H_2_O; 1.6 mg/l, ZnSO_4_·7H_2_O; 1.4 mg/l), CaCl_2_; 0.03%, urea; 0.03%, and MgSO_4_·7H_2_O; 0.02%, pH 5.5.

### Fungal Strains

In our previous work [43], wild filamentous fungi were isolated from niche substrates in the Fez-Meknes region, and their lignocellulolytic potential was profiled. Four strains were selected for this study, namely: *Penicillium crustosum* (ON180706), *Fusarium nygamai* (ON180709), *Trichoderma capillare* (ON180726), and *Aspergillus calidoustus* (ON180752). They were molecularly identified with the nuclear ribosomal Internal Transcribed Spacer (ITS) universal barcode. Species identification was refined using RNA polymerase II subunit 2 (RPB2). RPB2 sequences were deposited in NCBI under codes (*OR604502*, *OR604503*, *OR604504*, and *OR604505*) respectively. The first two fungi originated from decaying wood, the third was a soil-borne isolate, and the last was an olive pomace colonizer. They were grown, maintained on PDA solid plates by sub-culturing, and stored at 4°C when not immediately used.

### Olive Stones Biomass

Olive stones (OS) were sampled directly from OLEA FOOD Company, the largest olive oil producer in the Fez Meknes region, which uses the two-phase extraction system. OS were collected in sterile bags, air-dried, ground using a Romer Analytical Sampling Mill (RAS, Romer Labs, Austria), sieved into a homogenous substrate using standard test sieves (≤ 500 μm, Retsch, France), and stored at 4°C until use in fermentations.

### Fermentation Systems

A one-week-old culture of each fungus was used to prepare the inoculum. 10 ml of sterile distilled water with 0.01% Tween 80 was poured into the Petri dish to prepare a rich spore suspension. The inoculum solution was recovered, and its concentration was adjusted to 10^7^ spores/ml using a Neubauer counting chamber. Each flask was supplemented with 10^7^ spores of each fungal species separately. OS were used as the sole carbon source, and a substrate load of 5 g per flask was selected for all fermentations. Three fermentation systems were tried in our study to assess their effect on the production of lignocellulolytic enzymes by selected fungi. Namely, Solid-State Fermentation (SSF), Submerged Fermentation (SmF), and Sequential Fermentation (SF) were used. All flasks were incubated at 28°C for two weeks in complete darkness and static mode. All fermentations were conducted in duplicate.

**Solid-State Fermentation (SSF)** Each flask was supplemented with 5 g of OS as stated before. Moisture was adjusted to 75% by adding the liquid medium of Mendels & Sternberg. After sterilization and cooling, 10^7^ spores of the four strains were added aseptically and separately. Every 48 h, proteins and extracellular enzymes were extracted by adding 50 ml of distilled water to the solid medium, shaking at 180 rpm for 1h at room temperature, filtering using Whatman No. 1 filter paper, and then centrifuging at 10,000 ×*g* for 20 min at 4°C. The supernatant was used for proteins and enzyme activities’ quantification according to standard protocols described in Section 2.5.

**Submerged Fermentation (SmF)** Two hundred fifty milliliters (250 ml) flasks with a liquid working volume of 100 ml were prepared, where OS biomass was the sole carbon source. As for other fermentation systems, 10^7^ spores of each fungus were separately inoculated, and the flasks were incubated in static mode for two weeks. At every sampling point, the liquid broth was recovered, filtered, and centrifuged using the same conditions as above. The supernatant was used for proteins and enzyme activities’ quantification according to standard protocols described in Section 2.5.

**Sequential Fermentation (SF)** Sequential fermentation consisted of two stages. The first was a SSF pre-culture period lasting for 2 days and conducted as described above. Afterward, a transition to SmF was done by aseptically adding 100 ml of sterile liquid medium. Growth conditions were kept the same as for other fermentation systems, and the supernatant was similarly prepared and used for analytical purposes.

### Analytical Methods

**Olive stones’ characterization** The olive stones’ biomass was characterized for its composition and physicochemical properties. Moisture content was measured using a moisture analyzer (MD series, Japan). The insoluble ash content was determined with the traditional method, and the fiber content was quantified as three consecutive fractions using the standard protocols of Van Soest *et al*. [45]. Neutral-Detergent Fiber (NDF) was estimated by treating the sample with a neutral detergent solution to eliminate soluble sugars, starch, and pectins. The remaining residue is composed of hemicellulose, cellulose, and lignin. It was then treated with an acid-detergent solution to solubilize the hemicellulosic fraction and quantify the Acid-Detergent Fiber (ADF). The remaining solid fraction, which contains cellulose and lignin, was afterward treated with a concentrated sulfuric acid solution to solubilize cellulose and quantify the Acid-Detergent Lignin (ADL). All analyses were performed in duplicate. Hemicellulose, cellulose, and lignin contents were determined using the following formulas:

Hemicellulose = NDF – ADF (1)

Cellulose = ADF – ADL (2)

Lignin = ADL – Insoluble Ash (3)

### Evaluating the enzymatic potential of fungi

Lignocellulolytic index

As described by Pointing [46], an in-plate colorimetric method was used to assess endoglucanases (EG), lignin peroxidases, and manganese-dependent peroxidases (LiP and MnP), and laccase (Lac) enzymes’ production, respectively. Briefly, fungi were first grown on a solid medium containing the specific enzyme substrate at appropriate concentrations: 2% CMC for EG, 0.01% Azure B for LiP and MnP, and 0.1% ABTS for Lac. The cultures were monitored over 10 days, and positive enzyme production results were visualized as a yellow to dark orange halo zone around the colony for the EG (after staining with congo red and destaining with 1 M NaCl), a clearing zone for LiP & MnP, and a green coloration formation for Lac. Activity level was determined using the Enzyme Index (EI), calculated by dividing the halo diameter by the colony diameter [47]. Trials were performed twice to ensure accuracy.

Cellulolytic activities

Cellulolytic activities were assessed in three primary components as described by Ghose [48]: total cellulase activity using the Filter Paper Assay (FPA), endoglucanase activity using CMC as a substrate, and β-glucosidase activity using cellobiose. Briefly, enzyme extracts were buffered in 50 mM Na-citrate (pH 4.8) and supplemented with the specific substrate for each enzyme activity: strips (1 × 6 cm) of 1^st^ grade Whatman filter paper, 2% CMC and 50 mM cellobiose for FPA, EG and BG activities, respectively. The reaction tubes were incubated at 50°C for 1 h (FPA) or 30 min (EG and BG), supplemented with 3 ml of modified dinitrosalicylic acid (DNS) reagent [49], incubated at 100°C for 15 min to stop the enzymatic reaction and enable color formation, and then cooled in an ice bath. Reducing sugars liberated during saccharification react under heat with DNS to form a yellow to dark orange color. Hence, they were quantified as D-glucose equivalent at 540 nm, and enzyme activities were calculated. One IU/ml is defined as the amount of enzyme capable of catalyzing the conversion of 1 μmol of substrate per minute under reaction conditions.

Ligninolytic activities

According to Hariharan and Nambisan [50], ligninolytic enzymes were measured as Lac, LiP, and MnP activities using the colorimetric method. Lac activity was monitored by following the oxidation kinetics of ABTS over time at 436 nm. Enzyme supernatant (0.6 ml) was buffered in 0.3 ml Na-citrate pH 4.5, and 0.1 ml of 0.3 mM ABTS was added to start the reaction. For LiP activity, enzyme extract was buffered in 0.1 M of Na-citrate pH 3, and 1 mM of VA was added as the enzyme substrate. The reaction was initiated by H_2_O_2_, and the change in absorbance was monitored at 310 nm. Finally, MnP activity was measured at 610 nm by mixing enzyme extract, 0.1 M Na-citrate buffer pH 5, 250 mM Na-lactate, 2 mM manganese sulfate, 0.5 % bovine serum albumin, 0.2 mM H_2_O_2_, and 0.1% phenol red as the substrate. The enzymatic activities were calculated from the generated curves and expressed in IU/ml, as defined earlier.

**Proteins quantification** Proteins were quantified to investigate the correlation between their concentration and enzyme activity, which may provide information on the dynamics of enzyme production and secretion, allowing for the assessment of enzyme performance independently of total protein concentration by determining the specific activity targeted in our perspective work. The assay was performed using the Lowry at a wavelength of 750 nm. Bovine serum albumin was used for the standard curve, with concentrations ranging from 0 to 100 μg/ml [51].

### Experimental Methodology and Statistical Analysis of Data

A mixed-level full factorial design was conducted to assess the effect of the fungal strain and fermentation system on the expression of proteins, cellulolytic, and ligninolytic enzymes. Data were collected over time and expressed as mean ± standard deviation. ANOVA was used to test the different factors and their interactions. The relationships between quantitative variables were evaluated by the correlation method using the open-source R software [52]. Differences with a *p* < 0.05 were considered significant for all statistical tests, and graphical presentations were dressed using Excel.

## Results and Discussion

### Chemical Composition of the Olive Stones

The growth of fungi and their enzyme production is influenced by substrate complexity and composition, which can either enhance or inhibit enzyme activities [53]. Therefore, a chemical analysis was carried out to characterize the substrate selected for this work. Table 1 shows that OS exhibited a low moisture content. The residues were obtained from an olive oil production company that utilizes a two-phase system for processing. After being separated from the olive pulp and the extracted residual oil, the by-product undergoes a drying process. The reduced moisture content is beneficial for long-term preservation, as it minimizes microbial damage [54]. Additionally, the ash content found in the residues can provide a favorable environment for fungal growth.

With a cellulose content of 32.10%, the LC waste of this study demonstrates a considerable ability to stimulate cellulase activity. The presence of lignin, accounting for 24.07%, is linked to cellulose hydrolysis accessibility. Nait Mbarek *et al*. [41] described the chemical composition of the same by-product from a previous year as containing 37.73% acid-insoluble lignin with a low content of acid-soluble lignin. Hemicellulose also showed a significant percentage of 27.17%. This polysaccharide was reported to play a role in limiting enzymatic hydrolysis by interacting with cellulose and lignin, thus hindering cellulase access to cellulose. Hence, reducing the levels of lignin and hemicellulose in the biomass enhances the abundance of polysaccharides easily degradable by microorganisms [55]. Additionally, OS was reported to contain numerous bioconstituents, mainly phenols, fats, proteins, and free sugars. Phenolic compounds are among the most interesting constituents of olive-derived products. Tyrosol, hydroxytyrosol, oleuropein, Nüzhenide, and verbascoside are the predominant phenols reported in the literature [5].

Pretreatments can be applied to improve substrate amorphism and porosity by removing these structural compounds or reducing their recalcitrance [55]. The composition of the LC waste used in this study can be deemed suitable for diverse applications, capitalizing on both bioactive constituents and LC content. Additionally, this biomass is cost-effective, and its collection contributes to waste management, rendering it a feasible and sustainable alternative.

### Kinetics of Lignocellulolytic Cocktail Production

Considering the diversity in growth rates and enzyme secretion patterns displayed by the fungal strains, the fermentation period emerged as a crucial element that considerably influenced the synthesis of lignocellulolytic enzymes. The systematic monitoring of enzyme activity over time was used to determine the optimum incubation period for each strain cultivated under the different culture methods.

**Fig. 1:**
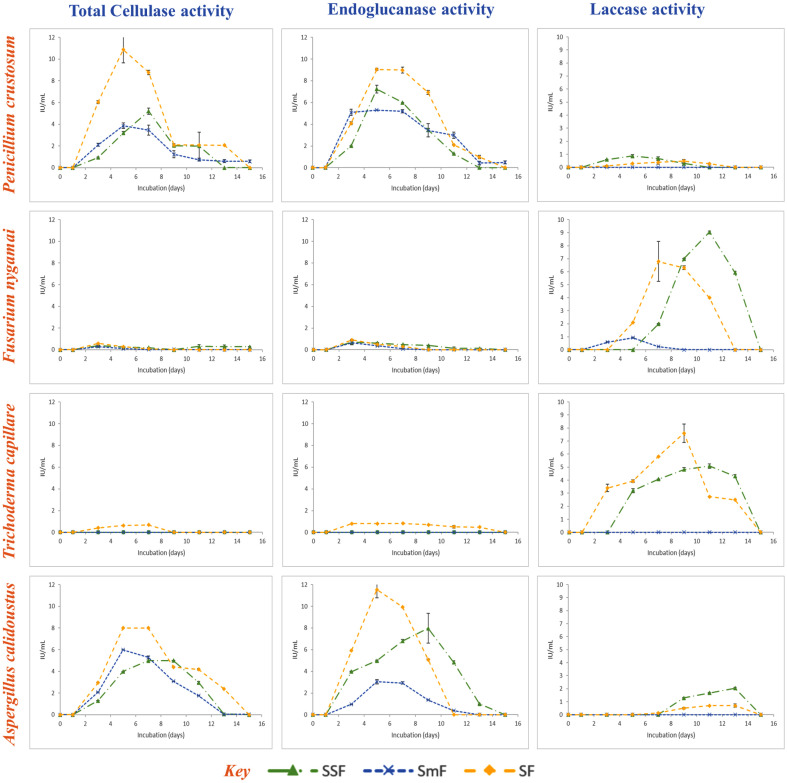
Kinetics of the cellulolytic and ligninolytic enzymes production under three fermentation modes. *Penicillium crustosum*, *Fusarium nygamai*, *Trichoderma capillare*, and *Aspergillus calidoustus* were cultivated during 15 days of dark incubation without agitation, and olive stones were used as substrate. Experiments were carried out in constant temperature (28°C), inoculum size (10^7^ spores), moisture content (75%), and pH (5.5) conditions.

**Fig. 2:**
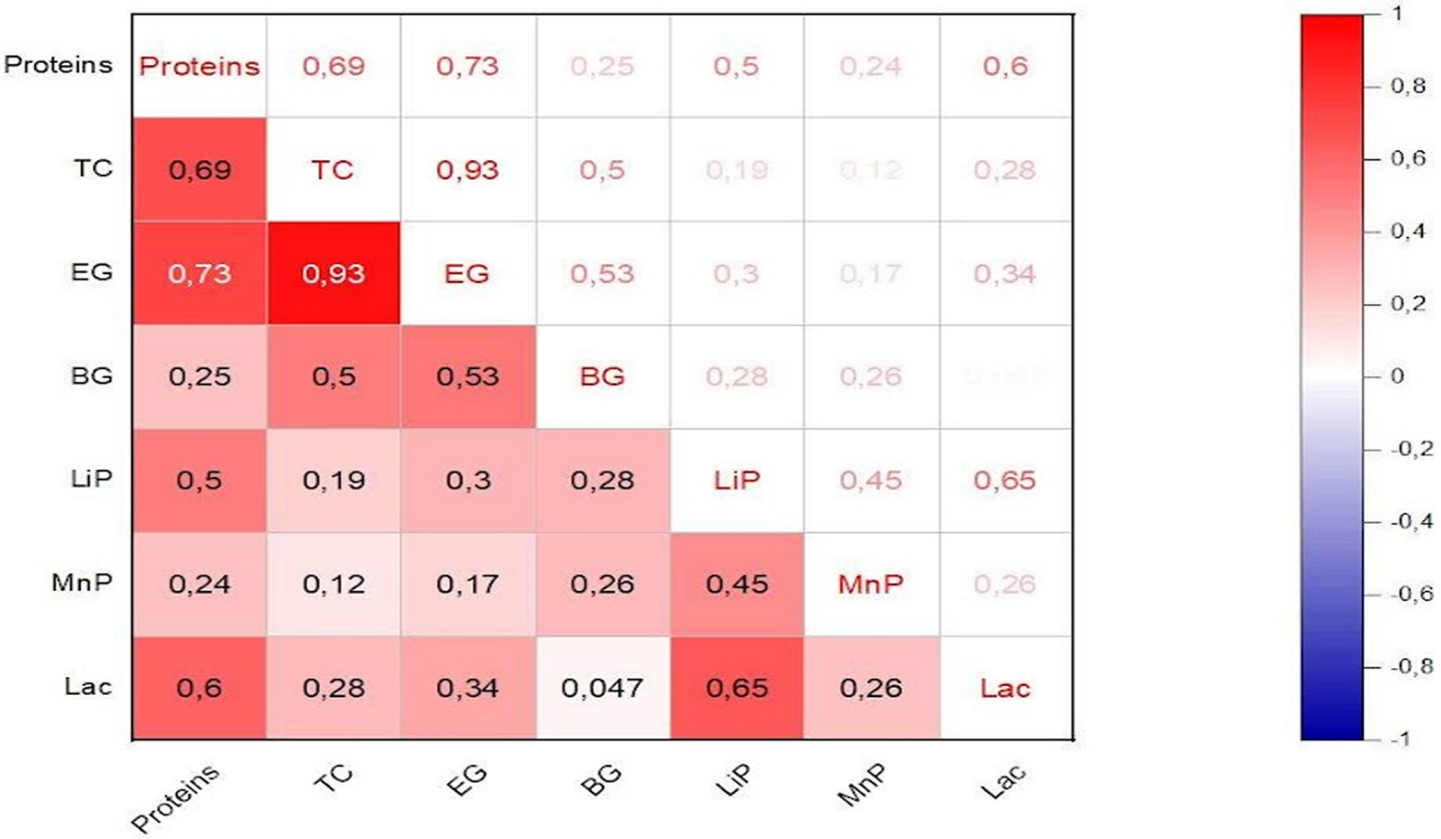
Correlation matrix between quantitative variables at a confidence interval of 95%. The correlation coefficient is statistically significant if the *p*-value is < 0.05. Quantitative variables refer to Proteins, TC: Total cellulase activity. EG: Endoglucanase activity, BG: β-Glucosidase activity, LiP: Lignin Peroxidase activity, MnP: Manganese-dependant Peroxidase activity and Lac: Laccase activity.

**Fig. 3:**
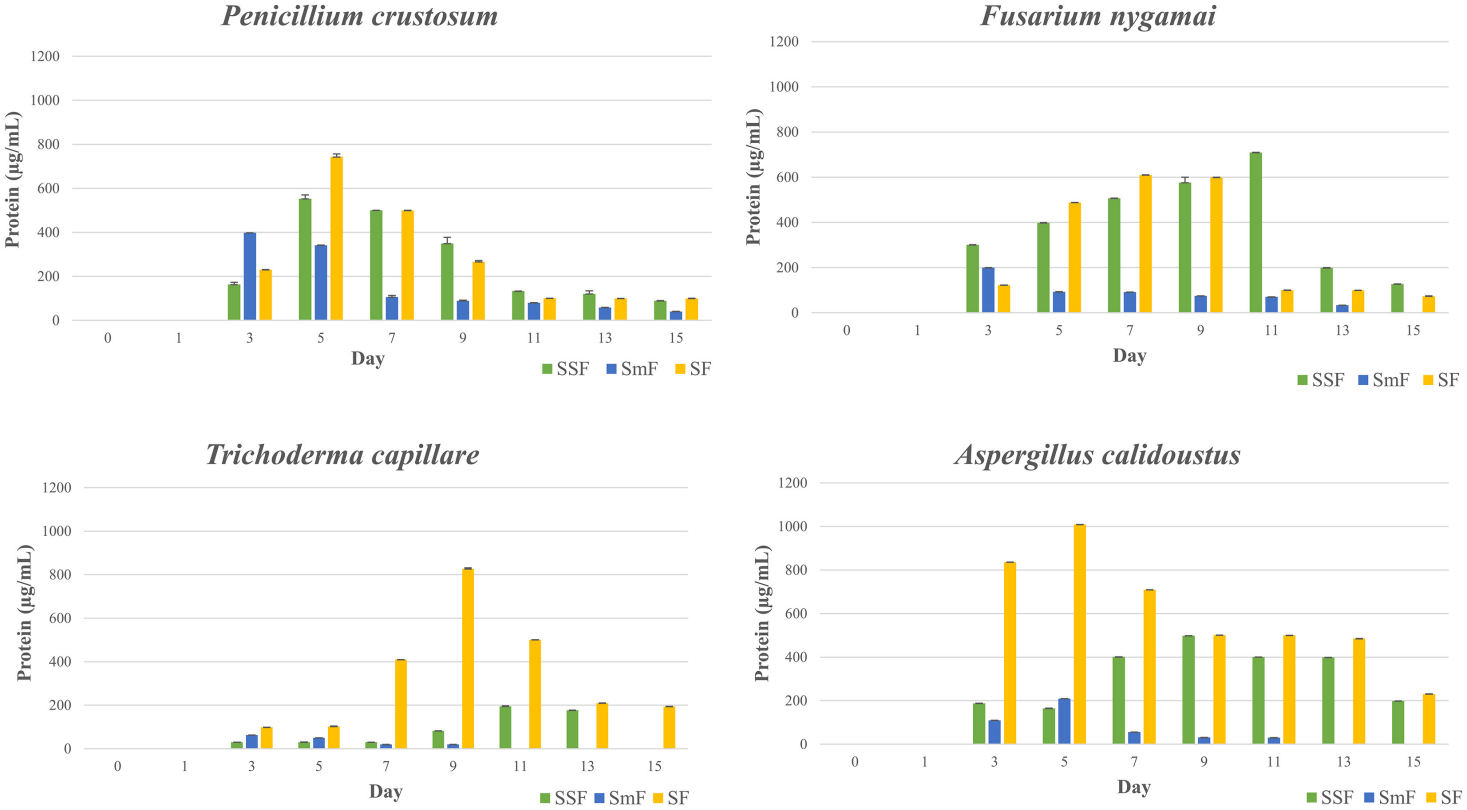
Protein concentration from crude enzyme cocktails produced by *Penicillium crustosum*, *Fusarium nygamai*, *Trichoderma capillare*, and *Aspergillus calidoustus* using olive stones as substrate during 15 days of dark incubation. SSF, SmF, and SF refer to solid-state fermentation, submerged fermentation, and sequential fermentation, respectively.

According to the statistical evaluations, the incubation period had a statistically significant impact on the expression of enzymatic activities (*p* < 0.05). Importantly, the four fungal strains showed remarkable differences in their production patterns of cellulases and ligninases (Fig. 1). Cellulolytic activity started after the third day of incubation and peaked between the third and ninth days. In contrast, the detection of ligninolytic activity was sometimes delayed, taking until the ninth day of incubation to be quantified and the thirteenth day of cultivation to reach peak enzyme activity, as for *A. calidoustus* under SSF and SF. On the fifteenth day of fermentation, enzyme activity was mostly lacking for the majority of strains, except for the TC activity recorded for *P. crustosum* and *A. calidoustus* under SmF, and for *F. nygamai* under SSF. Nevertheless, the activity levels observed were significantly lower than the optimum obtained during the earlier incubation period. Correlation analysis highlighted the relationship between protein production kinetics and enzyme activities. With a 95% confidence interval, positive correlations between protein production and the activities of TC, EG, and Lac were observed, with correlation coefficients of 0.69, 0.73, and 0.60, respectively. A strong positive relationship was also identified between TC and EG activities, with a correlation coefficient of 0.93 (Fig. 2). In terms of the maximum protein concentrations achieved, *A. calidoustus* stood out, reaching an impressive concentration of 1,009.1 μg/ml on the 5th day of incubation. This was followed by *T. capillare*, which achieved 827 μg/ml on the 9th day, and *P. crustosum*, reaching 743.2 μg/ml on the 5th day, both under sequential culture. *F. nygamai*, on the other hand, attained 709.4 μg/ml after 11 days of cultivation, under SSF (Fig. 3).

The results are consistent with the study conducted by Salgado *et al*. [56] which highlights the significance of fermentation time in the production of lignocellulolytic enzymes. It was shown that the production of endocellulase was favored by prolonged fermentation times. Typically, enzyme production increases with time until it reaches a peak, after which it might start to decline due to nutrient depletion or product inhibition [57, 58]. These two enzyme classes are known for their ability to work in synergy to break down the complex structure of lignocellulose. Cellulases target cellulose and hemicellulose, while ligninases break down lignin, removing the protective barrier and allowing cellulases easier access to their substrates. The initiation of cellulolytic activity on the third day may be linked to the time required for fungi to adapt to their environment and start producing enzymes. Substrate grinding and hydrothermal pretreatment (substrate sterilization before inoculation) can be important elements in improving the accessibility of LC materials to secreted enzymes. It was reported that the first pretreatment is advantageous as it allows reduction of cellulose crystallinity and polymerization extent, and increases the surface area available for enzymatic hydrolysis and mass transfer [59]. The latter also contributes to hemicellulose solubilization and lignin elimination [60]. The late expression of ligninolytic activity observed in our study is probably due to the complex regulatory mechanisms governing lignin degradation [61]. The positive correlations observed between protein production and enzyme activity are consistent with the idea that increased protein production may be linked to enhanced enzyme activity. According to the literature, higher protein concentrations often correspond to increased fungal biomass and active cellular processes, indicating favorable fermentation conditions. It could include both the enzymes produced by the fungi and the breakdown products of the substrate [62, 63].

Altogether, our research results underline the need to understand strain-specific differences and the influence of incubation time. They can contribute to a better comprehension of the dynamics of lignocellulolytic enzyme production, which is essential for optimizing industrial processes and biotechnological applications involving the degradation of LC biomass.

### Characteristics and Enzymatic Profile of the Fungal Strains

The choice of fungus can greatly impact the overall efficiency of lignocellulose conversion. For this reason, researchers often explore the diversity of microbial strains to identify potent lignocellulolytic enzymes producers. In our study, the four fungal strains, including *P. crustosum*, *F. nygamai*, *T. capillare*, and *A. calidoustus* were darkly cultivated under three different techniques. The two enzyme classes, cellulase and ligninases, of the four strains, were compared to the qualitative analysis performed to calculate the enzymatic index (Table 2). *P. crustosum*, as revealed through both qualitative and quantitative analyses, exhibited a remarkable enzymatic profile. It expressed a comprehensive range of enzymes, including TC, EG, BG, and Lacc, except LiP and MnP, which were not detected under the conditions used by the fungal species studied in this work. SF emerged as the most favorable culture technique for this strain, leading to the highest TC activity of 10.9 IU/ml (Table 3). *A. calidoustus* demonstrated consistency in the screened enzymatic activities, producing cellulases (TC and EG) and ligninases (Lac), with a maximum EG activity recorded of 11.5 IU/ml. *F. nygamai* showed that, in addition to TC and EG activities, an impressive laccase activity of 9 IU/ml, surpassing the other strains in this study. Unlike *T. capillare*, it displayed a distinct enzymatic profile. Its cellulolytic profile was not observed by the in-plate colorimetric method. However, TC and EG were quantified in its enzymatic extract. Notably, this fungus produced a significant amount of Lac activity reaching 7.6 IU/ml. Among the strains tested, *P. crustosum* stood out as the sole producer of BG under the conditions studied, with an optimum activity of 3.6 IU/ml recorded in submerged cultivation.

The findings collectively underscore the diversity of enzymatic capabilities among the four strains and highlight the importance of this fungal collection for specific biotechnological applications. Thus, in addition to the fermentation method used, the use of olive stones played a considerable role in enzyme induction, thanks to their LC-rich composition. The ability of *P. crustosum* to express multiple lignocellulolytic enzymes makes it a promising candidate for LC biomass degradation, especially with the identification of BG activity in its enzymatic extract. This result aligns with literature reports, as certain *Penicillium* species are recognized for their BG production. This enzyme acts in the hydrolysis of alkyl-, amino⁄aryl-D-glycosides, cyanogenic glycosides, disaccharides, and oligosaccharides as well as having transglycosylation activity, making it a promising target for a wide range of applications [64]. As well, the impressive Lac activity observed in *F. nygamai* and *T. capillare* is noteworthy and suggests its potential for applications requiring efficient lignin degradation. This enzyme, which belongs to the oxidoreductase group, is the most frequently used to degrade chemical pollutants. Its broad applicability stems from its low substrate specificity and its ability to perform monoelectronic oxidation on a wide spectrum of substrates within various complexes [65]. Additionally, the production of EG activity by *A. calidoustus* is a promising outcome as this genus fungus is recognized as a potential candidate for cellulase production. This enzyme is well suited to sectors involved in the production of biofuels, detergents, paper manufacturing, food and beverages, etc. [27]. As far as our knowledge extends, there are no previous reports in the literature regarding the specific enzyme activity of *F. nygamai* and *A. calidoustus*. Consequently, this study can serve as a valuable starting point for future research in this area.

Results between qualitative and quantitative assays can reveal significant differences, as the production of enzymatic activity is strictly linked to the culture conditions involved. Some of these include the composition of the culture medium, the carbon source used, the concentration of inoculum employed, the fermentation system applied, etc. As a result, agar plate screening can give a strong indication of the presence or absence of enzyme activity, and correlates favorably with the quantitative assay. However, the latter may need to be optimized to achieve better enzyme production, especially as the use of recalcitrant biomass represents a barrier to the accessibility of cellulolytic enzymes due to the presence of lignin [47, 55].

In this study, fermentation systems were operated under standard conditions as described previously. However, other factors are reported in the literature to influence the microorganisms' metabolism. In this sense, further studies can be carried out to investigate their effects on the production of the enzymes targeted in this work, such as agitation and light. Agitation represents the most crucial parameter in aerobic fermenters. On the one hand, it provides uniform temperature and gas conditions, and on the other, it enables better mass and heat transfer and facilitates uniform water addition, thus compensating for water lost through evaporation [66]. In terms of light, it plays an important role in various physiological regulatory mechanisms in fungi. It influences numerous signaling pathways [67]. Particularly for *T. reesei*, it has been described that the predominant cellulase transcription factor genes and about 75% of the genes encoding glycoside hydrolases display the potential for light-dependent regulation [68].

### Comparison between Fermentation Methods

The choice of fermentation method is significant because it affects both enzyme production and process efficiency. The culture method plays a crucial role in providing conditions for the adaptation and growth of microorganisms, making various fermentation techniques an attractive method for plant cell wall conversion. Compared to other approaches, the enzymatic arsenal secreted during the fermentation process is the most effective, thanks to the synergistic action of the enzymes, as each targets the product of the other [69].

In general, the results demonstrated (*p* < 0.05) that SF yielded the highest enzymatic activities for TC and EG, especially for *P. crustosum* and *A. calidoustus*, outperforming SmF, which proved more suitable for BG activity. Additionally, SSF exhibited promising ligninase activities, as could be seen with *F. nygamai*, which was able to express significant laccase activity (Table 3). Typically, SF showed notable advantages, mainly attributed to the integration of a solid-state pre-culture, creating an environment conducive to increased substrate-fungus contact. This phase facilitated the fungus's adaptation and growth, characterized by dispersed filamentous mycelia, compared to SmF which led to the formation of fungal pellets [30, 70]. With regard to previous studies, enrichment of the culture medium with glucose played a notable role in increasing cellulase production and promoting mycelial growth [71, 72]. Although SSF allows better substrate colonization and enzymatic degradation, low water activity can have an impact on growth and enzyme secretion [73]. Similarly, it was found in earlier research on enzyme production that different fermentation modes yielded divergent results depending on the fungus and biomass used. For example, *A. niger* expressed higher cellulase activities in SF compared to SmF with sugarcane bagasse [30], and better in SSF as opposed to SmF using coir waste as substrate [71]. In addition, the *Myceliophthora heterothallica* strain cultivated on wheat bran, sugarcane bagasse, and cardboard produced significant endoglucanase activity under SmF than under SSF [74]. In terms of ligninases activities, SSF was frequently reported to yield promising results. Particularly, white-rot fungi such as *Phanerochaete chrysosporium*, *Trametes versicolor*, and *Pleurotus ostreatus* are well-known for their efficient lignin degradation capabilities and production of ligninolytic enzymes, including lignin peroxidase, manganese peroxidase, and laccase [75].

It is important to note that the substrate composition should be carefully considered based on the specific enzyme to be produced, as it may contain substances that act as inducers. For instance, the utilization of residues that are abundant in lignin favored the production of lignin peroxidase [76]. On the other hand, employing wastes that are rich in cellulose stimulated laccase production [66, 77]. The findings show that enzyme production for any given cultivation method is critically dependent on the specific microorganism and substrate used.

Comparing different fermentation modes can deliver a valuable understanding of the efficiency and performance of different fungi and substrates. It allows the selection of the most productive technique for target enzyme production. Nevertheless, optimization steps are crucial to achieve maximum yields and provide recommendations for the scale-up of fungal-based LC bioprocesses.

## Conclusion

The use of LC biomass for the production of biofuels and other valuable products offers huge potential for significantly reducing the carbon footprint of present and projected energy needs. Enzymatic processing provides the benefit of being both exceptionally specific and cost-effective. Nevertheless, the cost of lignocellulose-degrading enzymes remains a major bottleneck to the development of efficient production methods. The efficiency of this process relies on key factors, mainly enzyme-producing microorganisms, the appropriate fermentation system, and the ideal substrate used. The results of the current study show that at *p*-value < 0.05, the different factors (strains, substrate, and culture conditions) significantly affect the production of lignocellulolytic enzymes. The four strains investigated revealed diverse and relevant enzymatic activities under the three fermentation methods. The results suggest that the strains studied require at least one solid phase to induce enzyme activity. In light of these results and with a view to improving the production of the enzymes sought, further studies are required, such as the application of pretreatments to increase the exposure of LC materials to the enzymes, followed by the optimization of fermentation conditions using response surface methodology (RSM). And to unveil the interest of this biomass in the generation of a molecular platform, the analysis of degradation products and the identification of relevant molecules generated could be considered. Eventually, this study provides an attractive pathway for the use of fungal biotechnology at the national level to produce enzymes of industrial interest from low-cost raw materials.

## Figures and Tables

**Table 1:** Chemical characterization of Moroccan olive stones. Data were expressed as mean ± standard deviation.

Component	Content (%)
Hemicellulose	27.2 ± 0.6
Cellulose	32.1 ± 0.6
Lignin	24.1 ± 1.6
Ash	2.8 ± 0.3
Moisture	10.1 ± 0.1

**Table 2:** Enzymatic Index of four wild filamentous fungi isolated from Morocco.

Fungi	Enzymatic Index (EI)		
	EG	LiP & MnP	Lac
*Penicillium crustosum*	2.4	ND	2
*Fusarium nygamai*	2	ND	3.3
*Trichoderma capillare*	ND	ND	2.8
*Aspergillus calidoustus*	2.9	ND	2.2

ND: Not detected.

**Table 3:** Effect of strain and fermentation mode on the optimum production of cellulolytic and ligninolytic enzymes produced by *Penicillium crustosum*, *Fusarium nygamai*, *Trichoderma capillare*, and *Aspergillus calidoustus*. Data were expressed as mean ± standard deviation.

Fungi	Fermentation mode	Prot	TC	EG	BG	LiP	MnP	Lac
*P. crustosum*	SSF	552.9 ± 16.8	5.2 ± 0.3	7.2 ± 0.4	1.5 ± 0.6	ND	ND	0.9 ± 0.1
	SmF	398 ± 0.1	3.9 ± 0.2	5.3	3.6 ± 0.8	ND	ND	ND
	SF	743.2 ± 12.7	10.9 ± 1.2	9 ± 0.1	2 ± 0.8	ND	ND	0.5 ± 0.1
*F. nygamai*	SSF	709.4 ± 0.9	0.4	0.7 ± 0.1	ND	ND	ND	9 ± 0.1
	SmF	199.8 ± 0.2	0.3	0.7 ± 0.2	ND	ND	ND	0.9
	SF	609.4 ± 0.9	0.6	0.9 ± 0.1	ND	ND	ND	6.8 ± 1.5
*T. capillare*	SSF	194.8 ± 2.3	ND	ND	ND	ND	ND	5.1 ± 0.2
	SmF	63 ± 0.1	ND	ND	ND	ND	ND	ND
	SF	827 ± 4.2	0.7	0.8	ND	ND	ND	7.6 ± 0.7
*A. calidoustus*	SSF	498	5	8 ± 1.4	ND	ND	ND	2 ± 0.1
	SmF	209.4 ± 0.2	6	3 ± 0.1	ND	ND	ND	ND
	SF	1009.1	8	11.5 ± 0.8	ND	ND	ND	0.7 ± 0.1
Fungus sig		***	***	***	***	---	---	***
Fermentation mode sig		***	***	***	*	---	---	***
Interaction Fungus-Fermentation sig		***	***	***	*	---	---	***

Prot: Proteins. TC: Total cellulase activity. EG: Endoglucanase activity. BG: β-Glucosidase activity. LiP: Lignin Peroxidase activity. MnP: Manganese-dependant Peroxidase activity. Lac: Laccase activity. SSF: Solid-State Fermentation. SmF: Submerged Fermentation. SF: Sequential Fermentation. ND: Not detected. Significance levels: *p < 0.05; **p < 0.01; ***p < 0.001; n.s = *p* > 0.05.
